# Patient-delivered tDCS on chronic neuropathic pain in prior responders to TMS (a randomized controlled pilot study)

**DOI:** 10.2147/JPR.S186079

**Published:** 2018-12-10

**Authors:** Francis O’Neill, Paul Sacco, Eleanor Bowden, Rebecca Asher, Girvan Burnside, Trevor Cox, Turo Nurmikko

**Affiliations:** 1 The Pain Research Institute, Faculty of Health and Life Sciences, Clinical Sciences Centre, University of Liverpool, Liverpool, UK, foneill@liverpool.ac.uk; 2 Cancer Research UK Liverpool Cancer Trials Unit, Liverpool, UK; 3 Department of Biostatistics, University of Liverpool, Liverpool, UK

**Keywords:** neuropathic pain, brain stimulation, motor cortex, M1, transcranial direct current stimulation, tDCS, transcranial magnetic stimulation, TMS

## Abstract

**Background:**

Successful response to repetitive transcranial magnetic stimulation (rTMS) of the motor cortex requires continued maintenance treatments. Transcranial Direct Current Stimulation (tDCS) may provide a more convenient alternative.

**Methods:**

This pilot study aimed to examine the feasibility of a randomized, double-blind, double-crossover pilot study for patients to self-administer tDCS motor cortex stimulation for 20 minutes/day over five consecutive days. Primary outcomes were as follows: usability of patient-administered tDCS, compliance with device, recruitment, and retention rates. Secondary outcomes were as follows: effect on overall pain levels and quality of life via Short Form-36 anxiety and depression via Hospital Anxiety and Depression Scale, and Mini-Mental State scores.

**Results:**

A total of 24 subjects with neuropathic pain, who had previously experienced rTMS motor cortex stimulation (13 with reduction in pain scores, 11 nonresponders) were recruited at the Pain Research Institute, Fazakerley, UK. A total of 21 subjects completed the study. Recruitment rate was 100% but retention rate was only 87.5%. All patients reported satisfactory usability of the tDCS device. No significant difference was shown between Sham vs Anodal (−0.16, 95% CI: −0.43 to 0.11) *P*=0.43, Sham vs Cathodal (0.11, 95% CI: −0.16 to 0.37) *P*=0.94, or Cathodal vs Anodal (−0.27, 95% CI: −0.54 to 0.00) *P*=0.053 treatments. Furthermore, no significant changes were demonstrated in anxiety, depression, or quality of life measurements. The data collected to estimate sample size for a definitive study suggested that the study’s sample size was already large enough to detect a change of 15% in pain levels at 90% power for the overall group of 21 patients.

**Conclusion:**

This study did not show a beneficial effect of tDCS in this group of patients and does not support the need for a larger definitive study using the same experimental paradigm.

**Trial registration:**

ISRCTN56839387

## Introduction

Stimulation of the primary motor cortex (M1) has been shown to be effective in the treatment of chronic neuropathic pain.^1^ Early studies of neuromodulation for relief of chronic pain demonstrated the benefit of direct electrical stimulation using electrodes surgically implanted on the surface of the brain.^2^,^3^ However, given the invasiveness of this technique and the risk of complications and expense, it has not been widely adopted. One promising area of research on the treatment of chronic pain involves applying noninvasive brain stimulation techniques, which may act to modulate abnormal processes associated with this condition. These techniques include repetitive transcranial magnetic stimulation (rTMS) and transcranial direct current stimulation (tDCS). Although the results for rTMS therapy in chronic pain appear promising,^4^,^5^ its clinical use is limited by the short duration of action, necessitating frequent repeat treatment sessions, thus raising issues of service capacity and patient compliance. Indeed, a more common practice is to use TMS as a screening method for invasive epidural motor cortex stimulation.^6^,^7^

TMS involves placing a coil over the scalp through which an electric charge is passed, generating a magnetic field, which can induce an electric pulse in the brain sufficient to activate cortical neurons. When appropriate patterns or trains of pulses are used, it results in modulation of activity of cortical circuitry,^8^ which can be excitatory or inhibitory. Importantly, rTMS can affect the excitability of brain regions distant to the target site through synaptic connections to functionally related brain regions.^9^ This is thought to explain why motor cortex stimulation has an analgesic effect in chronic neuropathic pain. tDCS does not directly activate cortical neurons but rather affects their excitability via modulation of resting membrane potential.^10^ Anodal stimulation increases excitability of affected neurons and cathodal stimulation reduces excitability.^11^ Studies of anodal tDCS of the M1 motor cortex in fibromyalgia, pelvic pain, multiple sclerosis, and cancer pain, and phantom limb patients have been mostly positive,^12^–^17^ while those in neuropathic pain as a result of spinal cord injury have been mixed.^18^–^23^

We wished to establish the efficacy of using a patent’s response to motor cortex rTMS as a screening method for tDCS designed for home-based use and to assess the feasibility and efficacy of using home-based tDCS therapy in two groups of neuropathic pain patients (responders vs nonresponders to rTMS).

The plan was to design a definitive trial to test whether tDCS over the M1 cortex reduces pain scores in subjects known to have previously gained relief from high-frequency rTMS of primary motor cortex. Our hypothesis was that rTMS responders would derive greater benefit from anodal tDCS than nonresponders. Furthermore, we postulated that there would be no difference between responders and nonresponders in pain relief from cathodal or sham tDCS.

As this study design includes the use of patient-delivered tDCS, a pilot study was necessary to examine the practicality of this approach.

The aims of this pilot study were to test whether tDCS over M1 area could be manageably delivered by patients or carers at home, and to test recruitment and retention rates for this study design.

The study design also collected outcome data on average weekly pain scores, quality of life via Short Form-36 (SF-36), anxiety and depression via Hospital Anxiety and Depression Scale (HADS), and Mini Mental State Examination (MMSE) to assess for cognitive changes. These assessments will form the primary and secondary outcomes of the full trial. These data will be used to inform sample size calculations for future study. Overall data will be used to identify challenges in the study conduct.

## Methods

### Study participants

All participants had previously taken part in a clinical trial examining effect of rTMS motor cortex stimulation in chronic pain.^24^ As subjects were recruited from this study pool, their response to previous treatment with rTMS for chronic pain was known.

#### Inclusion criteria

1) Age between 18 and 85 years old. 2) Diagnosis of unilateral neuropathic pain as defined by the International Association for the Study of Pain Special Interest Group on Neuropathic Pain.^25^ 3) Stable analgesic medication for prior month. 4) Average pain levels >4 out of 10 on numerical rating scale (NRS) for pain during the 1-week run-in phase, based on patient diary. 5) Willingness to take part in the study and ability to give consent. 6) Previously had a minimum of 5 sessions of rTMS for pain, and can be named as a “responder” or “nonresponder”. Responder: reporting a weekly average pain reduction of a minimum of 15% on an NRS of 0–10, following five rTMS sessions. This was chosen as the minimally important change in pain scores as defined by the Initiative on Methods, Measurement, and Pain Assessment in Clinical Trials consensus recommendations.^26^ 7) Subjects were required to have had pain for >6 months.

#### Exclusion criteria

1) Pain of other origin, for example, musculoskeletal pain as this could interfere with the reporting of the neuropathic pain being targeted. 2) Metal implants/coils/electronic devices. 3) Drug or alcohol abuse. 4) Pregnancy. 5) Psychiatric or psychological disorders. 6) Epilepsy. 7) Inability to understand instructions or operate equipment. 8) High-dose opioids. 9) Uncontrolled medical conditions (eg, active cancer, uncontrolled renal, pulmonary, or cardiac disease, etc).

The study was carried out at the Pain Research Institute, Clinical Sciences Center, Fazakerley, Liverpool, UK.

This study was conducted in accordance with the Declaration of Helsinki. Ethics approval was obtained from the national ethics committee (NRES REC reference 12/EE/0315), and all subjects gave informed written consent to participate in the study.

### Study design

A randomized, double-blind, double-crossover design was used so that all subjects participated in both anodal and cathodal active treatments (tDCS) and sham treatment (Trial registration: ISRCTN56839387). The treatment sequence was randomized and counterbalanced and the subjects and the response assessor were blinded to it.

#### Assessment of recruitment and retention rates

**Figure 1:**
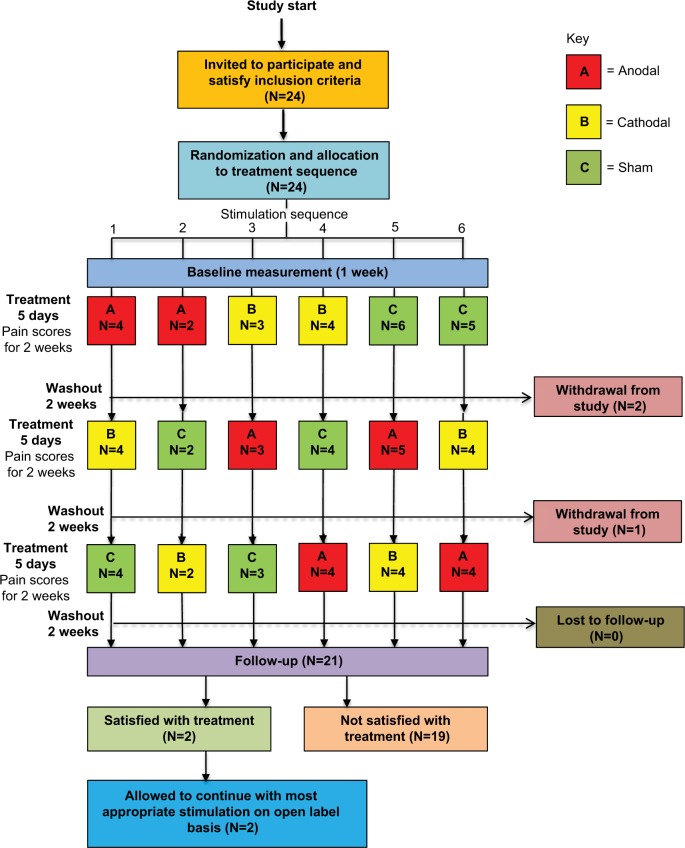
CONSORT Flowchart. **Note:** Trial design, including participant flow and dropouts, showing 24 subjects recruited and randomized with a total of 3 withdrawals from the study, giving 21 subjects at follow-up.

Assessment of pain and other pain-related variables occurred at initial assessment, for 14 days starting at and including each 5-day period of intervention, and at 4-week followup (after a minimum 2-week period for washout). Stable medication use before and during the trial was required. The randomized crossover design is shown in Figure 1 (Consort Flowchart^27^,^28^). The study protocol has been published previously.^24^ No changes to the protocol were made.

### Interventions

We wished to optimize patient selection to those patients who had previously undergone mapping of the motor cortex using brain navigation TMS localization, and therefore, had a known degree of cortical reorganization, with optimal coordinates for each participant available to guide the placement of tDCS electrodes on the scalp.

#### Identification of motor cortex stimulation site

As subjects had previously been enrolled in a TMS study evaluating the impact of target location on pain relief, they had undergone MRI of the brain and mapping of the motor cortex.^24^ All participants had undergone a trial of two separate locations within M1, contralateral to their pain, and the location of the active tDCS electrode was placed over the site at which the best rTMS response was obtained.^24^

#### tDCS treatment

tDCS used was similar to that described previously^18^ but subjects were instructed how to administer the stimulation themselves. For each active treatment, one 20-minute session was delivered each day for five consecutive days. A 4-week period occurred between each treatment type (active and sham). Direct current was applied to the scalp by a commercial tDCS unit using a pair of saline-soaked sponge-covered surface electrodes (HDCstim, Newronika s.r.l., Milan, Italy). This system has a maximum stimulation setting of 1.5 mA and proprietary-sized electrodes (25 cm^2^, 5 × 5 cm). A current of 1.4 mA (30 second ramp on) was delivered, giving a current density of 0.056 mA/cm^2^, which is comparable to previous studies.

During active tDCS treatment, subjects received stimulation of the primary motor cortex (M1). For anodal stimulation, the anode electrode was placed over the previously identified “hotspot” during TMS mapping and the cathode electrode over the contralateral supraorbital area. For cathodal stimulation, the electrodes were reversed. Stimulation was applied to the opposite hemisphere to the side in which the dominant neuropathic pain was felt.

#### Patient-administered stimulation

At the initial visit, measurements were taken for individually fitted headband locators to facilitate electrode placement. Once fitted, subjects were shown how to position the headbands reproducibly and asked to demonstrate this to the investigator. Photographs and measurements were taken by the patients in case of the need for reference at home. Electrode placement was checked at the beginning of each treatment block.

Patients were administered a constant current of 1.4 mA for 20 minutes daily for 5 days at the same time of the day. All subjects were asked to remain non-active during this time and engage in either light reading/watching television or listening to the radio.

#### Assessment of usability of device

Patients were asked to record the ease of use of their tDCS device setup and report if they had any difficulties with electrode placement or device use. Each device kept an internal record of the number of times the device was used and an exact record of the date, time of day, and duration the device was used for. It also recorded the current and average resistance during each stimulation. This allowed the investigator to track the use of the device.

#### Assessment of device compliance

##### Interrogation of tDCS devices

Individual stimulators were interrogated after each 5-day period of use to check correct stimulation delivery. The device records the number of stimulations delivered, the time and date of stimulation, and the stimulation parameters, including current strength, duration of stimulation, and resistance of circuit in ohms. It also recorded if any error occurred during stimulation, eg, if stimulation was aborted. This allowed full assessment of device compliance with each patient and treatment block.

##### Sham intervention

For sham stimulation, the electrodes were placed in the same positions as that for anodal M1 stimulation; however, a constant current of 1.4 mA was delivered only for 5 seconds (30-second ramp on). This sham delivery is pre-programmed into the device and automatically stops at the correct time period. The settings on the device visible to the patient are indistinguishable from active treatment settings. A countdown timer on the display panel will continue to count down over 20 minutes and the patient then removes the device. During active tDCS treatment, subjects typically report tingling sensations under the electrodes, which rapidly fade.^29^ Our sham intervention was, therefore, designed to provide an initial period of tingling so that similar sensations were perceived during active and sham tDCS protocols. This sham protocol has been used by previous investigators.^18^,^30^

### Randomization and blinding

Subjects were randomized in two ways. First, each stimulation device was assigned a random treatment order. Second, each subject was assigned to a random device number. Both these tasks were completed by a researcher (unrelated to the study) by picking the folded paper containing the treatment order/subject order from an urn. This order was written down as a master key and divided into two study keys. A research assistant (not otherwise related to the study) set the stimulation parameters as indicated by the first study key, and was aware of the treatment order each device was programmed for but was unaware which subject received each device.

The investigator was aware which device to give to each subject by following the second study key but was unaware of the treatment order programmed in that device. The master randomization key was kept in a sealed envelope.

Subjects were not directly informed that a sham stimulation was being used. Rather they were told that three different types of stimulation treatments would follow each other, some of which may or may not feel different.

Only at the end of the study were subjects informed that a sham stimulation was one of the treatments. At this time, they were asked verbally if they were able to guess the order of stimulation. Their guess was checked against the randomization key.

## Assessments

### Primary outcome measures

#### Usability of patient-administered tDCS

The ease of use of the tDCS setup for patient administration was assessed by a patient experience questionnaire. This included four questions: 1. Did you feel the device was easy to use? 2. Did you have any problems using the device? 3. Did you have to contact a member of the research team for advice about using the machine while at home? 4. Did you have to abandon using the device for any reason?

#### Patient compliance with device

As patients may have felt the device was easy to use but still have used it incorrectly, compliance with the device was assessed. This was done by a combination of device interrogation to discern the timing, number, and duration of stimulations delivered during use and if any errors had occurred. Also, at return appointments, patients were asked to demonstrate the positioning of the electrode placements to ensure that they were complying with the correct anatomical positioning.

#### Recruitment and retention

A record was kept of the number of patients recruited and the date they joined the study. Also, the number, timing, and reasons for any patient withdrawals from the study were documented.

### Secondary outcome measures

Effect on overall pain was assessed on an 11-point (0–10) NRS questionnaire. Patients were asked to complete these at home each night based on the average pain scores over the previous 24 hours. Pain diaries were returned at the next patient visit.

Subjects completed the following questionnaires at baseline and at the end of each treatment period: SF-36 to assess quality of life, HADS to assess anxiety and depression, and MMSE to assess for cognitive changes. Patients were also asked to complete a questionnaire regarding any difficulties encountered in using the device during the trial.

### Statistics

#### Sample size

For this pilot study, the sample size was based on an opportune sample of 24 patients who had previously undergone rTMS and expressed willingness to take part in the study. No formal power calculations were carried out prior to the study. Results from this study have been used to calculate a definitive sample size from the recorded effect size and SDs of the observed data. This is reported in the discussion at the end of this paper.

#### Statistical analyses

Simple descriptive statistics were used to describe the primary outcomes and adverse events.

To investigate the effect of the three modes of stimulation on the secondary outcomes of interest, a linear mixed effects model was used. The mode of tDCS, treatment period, responder group, and time were all regarded as fixed effects, and the patient (nested in sequence) as the random effect. Three time points were used when measuring daily pain intensity; days 1, 5, and 14, which corresponded to the beginning and end of treatment, and the end of the observation period, respectively. The mean of the 7-day baseline pain diary was used as baseline for the first mode of treatment. As a baseline pain diary was not completed before each tDCS treatment period, the pain score recorded on the first day of the observation period was taken as the baseline measurement for the second and third treatment. A one-way ANOVA test was implemented on these measurements to ensure that they did not differ significantly between treatments. As the secondary outcomes – HADS and SF-36 – were only measured once for each treatment, a time component was not included as a fixed effect when analyzing them.

For sensitivity analysis, baseline and clinical measurements were added one by one to the model and those that altered the results significantly were included. A treatment by period interaction was investigated to see whether there was a difference in pain according to the sequencing of treatments.

The association between tDCS and rTMS responders was investigated using Fisher’s exact test. A tDCS responder was classified as a pain reduction from baseline of at least 15%.

All analyses were performed using Stata version 13 (StataCorp. 2013. *Stata Statistical Software: Release 13*. College Station, TX, USA: StataCorp LP).

## Results

### Patient demographics

A total of 24 subjects (nine female, 15 male) with unilateral neuropathic pain were recruited for this study between October 2012 and August 2014. Of those recruited, 13 (54%) were prior responders to rTMS treatment. The mean duration of pain was 8.04±4.9 years (Table 1).

### Primary outcomes

#### Recruitment and retention rate

The target number of patients was recruited within the allotted time period. Although the study required several attendances at the research unit and also required multiple treatment sessions, only three patients (12.5%) dropped out, with a retention rate of 87.5%.

#### Losses and exclusions

One subject was excluded from the analysis as the patient failed to complete/return any pain diaries within the time frame of the study; the subject completed the randomized treatment. Two patients withdrew from the study; one withdrew after the first sham trial due to an exacerbation of pain and one withdrew after one sham trial and one failed cathodal trial, reporting difficulty in accommodating treatment sessions with daily schedule. One subject returned an incomplete pain diary for the sham trial and one subject returned an incomplete diary for the cathodal trial and failed to return one for the anodal trial.

#### Usability of patient-administered tDCS

All patients reported the device was easy to use with a general appreciation of the convenience of home-based stimulation. This reflects the simplicity of this particular model of tDCS device with a simple on/off switch and start button. Positioning of headbands was not considered a problem. All subjects said that the positioning marking on the headbands was easy to follow and positioning was easy to replicate. Two subjects with single limb paralysis even reported being able to position the headbands one handedly, although they usually received help from a relative for speed. One subject reported that the headband would slip during use, which required readjustment. One subject who did not return any pain diaries during the study did not report any problems using the device. Another subject felt that although the device itself was easy to use, the time required for treatment sessions, that is, 20 minutes for stimulation and ~10 minutes setup time was difficult to fit in with daily activities.

The main issue affecting compliance was that if there was unsatisfactory contact between the electrode and skin, then the current circuit was broken and the device would abort the stimulation. This was usually easily rectified by re-wetting of the electrode sponge; however, the patient would need to start a different stimulation session. In practice, when this happened, an extra stimulation was delivered if the patient noticed but if it went unnoticed, an aborted stimulation was recorded on the device log.

Seven patients (29%) reported that they had to contact the research team for advice regarding this issue. All issues were resolved via a telephone conversation. Two patients (8%) did not notice a signal failed stimulation; this was subsequently identified on interrogation of the device log. No subjects had to totally abandon using the device.

#### Patient/device compliance

The actual number of stimulations self-administered by subjects per trial is shown in Table 2. In total, 72 trials comprising of five stimulations per trial were planned over the course of the study. Of these 72 trials, six were not completed due to three subject withdrawals and ten were completed differently from the protocol. Of the ten trials not completed as directed, four of the five stimulations were delivered in six trials and in one trial, three stimulations were delivered. In the remaining three trials, extra stimulations were recorded due to either a failed or aborted activation of the device and the patient subsequently delivering further stimulations to reach the full five stimulations as per protocol. Only two subjects guessed the randomization sequence correctly, while most said that they would be unable to guess.

#### Adverse events

A total of 16 (62%) subjects reported tingling of the forehead/scalp and 12 (50%) reported redness after treatment, both during active and sham stimulations. Two (8%) reported tiredness. One subject reported a sharp increase in pain after sham stimulation and withdrew from the study. Five (20%) subjects reported headache following treatment, which lasted for ~2 hours and occurred in both active and sham stimulations. Two (8%) patients reported an exacerbation of parathesia experienced in the affected area at 4-week follow-up.

### Secondary outcomes

#### Overall pain intensity

Numerical pain rating scores for each treatment over time are summarized in Table 3. Comparison of baseline pain scores showed no significant difference between the three treatment arms (one-way ANOVA, *P*=0.9941).

**Figure 2:**
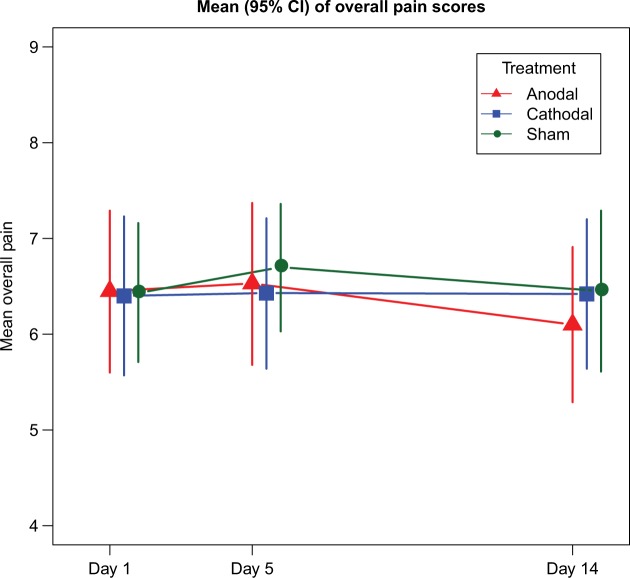
Effect of tDCS on overall pain scores. **Note:** X-axis= time in days from start of treatment session. Y-axis= mean pain scores on 11-point (0–10) numerical pain rating scale with (95% CIs). No significant differences were observed between Sham, Anodal, or Cathodal stimulations. **Abbreviation:** tDCS, transcranial direct current stimulation.

A linear mixed model was fitted to the data and the treatment-by-day and treatment-by-period interactions were investigated. There was no significant difference in overall pain between treatments (Figure 2); Sham vs Anodal (–0.16, 95% CI: –0.43 to 0.11, *P*=0.43), Sham vs Cathodal (0.11, 95% CI: –0.16 to 0.37, *P*=0.94), or Cathodal vs Anodal (–0.27, 95% CI: –0.54 to 0.00, *P*=0.053). No significant difference was seen between mean pain scores on day 1, 5, or 14 for any treatments; day 5 vs day 1 (0.13, 95% CI: –0.13 to 0.38, *P*=0.625), day 14 vs day 1 (–0.04, 95% CI: –0.29 to 0.22, *P*=0.788), and day 14 vs day 5 (–0.16, 95% CI: –0.42 to 0.10, *P*=0.216). No carryover effects were present.

#### HADS and SF-36

Mean values of anxiety and depression scores are shown in Table 4.

#### Anxiety

Effects on anxiety and depression scores are summarized in Table 5. Anxiety was reduced in treatment period three regardless of treatment modality (difference 1.20, *P*=0.014) and also showed a greater reduction with increased number of stimulations (–1.45, 95% CI: −2.34 to –0.56, *P*=0.002). However, the effect from the number of stimulations could be spurious as 85% of the trials recorded five simulations. There was an imbalance in mean levels of anxiety within the sequences. The average anxiety for sequence Cathodal–Anodal–Sham was much higher than that for the other sequences at baseline and throughout, while the mean for sequence Sham–Anodal–Cathodal was lower than the rest.

#### Depression

No differences were seen in depression between treatment modalities.

#### SF-36

Analysis of effect on two summary domains of patient-reported health outcomes in the SF-36 questionnaire Physical Component Summary (PCS) and Mental Component Summary (MCS) showed that tDCS treatment had no effect on PCS or MCS. An increase in the number of stimulations was associated with an increased MCS (4.69, 95% CI: 1.07 to 8.31, *P*=0.013).

#### Proportion analysis

The three treatment groups were divided into two categories according to the reduction in pain on the fourteenth day and Fisher’s exact test was used to find any association between rTMS and tDCS responders. No relationship was found.

## Discussion

This study, in which a cohort of chronic pain patients who had previously undergone treatment with rTMS were trialed with anodal, cathodal, and sham tDCS stimulation, failed to demonstrate a beneficial effect of tDCS in this group. This suggests that even in patients who respond well to rTMS to the motor cortex, substitution of TMS for tDCS motor cortex stimulation is not as suitable as anticipated, at least not in this group of patients and with this electrode configuration. It has, however, demonstrated that recruitment, retention, and compliance within this study design are feasible.

### Limitations

#### Compliance

Patients were asked to perform self-administered tDCS stimulation at home. As stimulation was not directly observed by a researcher, variation in electrode placement may have taken place. However, when asked to complete a questionnaire on ease of use of the tDCS device, none of our subjects reported placement of electrodes to be an issue. All subjects were individually fitted with measured headbands to securely hold electrodes and shown how to reproducibly locate them.

The analgesic effect of tDCS may be cumulative with greatest effect after 5 days of treatment.^12^,^18^ We cannot exclude that the seven trials in which fewer than five consecutive days of tDCS stimulations were delivered could have reduced any overall effect, but no clear trend was observed in the study.

### Generalizability

Is there a possibility that prior exposure to rTMS could have affected subsequent response to tDCS? While interaction of these two different forms of stimulation has been documented,^31^,^32^ these findings have been in relation to tDCS priming prior to TMS. Furthermore, given the short duration of effect of tDCS, any priming phenomena are only demonstrable if the two are combined within a short timeframe.

Long-lasting changes in cortical excitability following TMS are protocol-dependent but at best their duration lasts up to 2 weeks.^33^ Permanent changes have not been demonstrated.^8^,^34^ Given that all subjects recruited to this study had not received TMS treatment for at least 3 months, a priming effect is considered by the authors as unlikely.

Regardless of the effect of rTMS on neuronal priming or response, pain scores in two of these subjects were lower after finishing rTMS therapy and remained lower throughout their inclusion in this study. Perhaps this may have represented a flooring effect and the limit of the possible treatment effect that could be expected in these patients.

All the subjects included in this study had experienced pain for at least 3 years. Moreover, these were patients who had been through at least one other clinical trial assessing treatment for intractable chronic pain, meaning that they had failed multiple pharmacological therapies before entry into the present study. This group may represent a particularly treatment-resistant group of patients with pain. Indeed, their mean numerical pain rating scores were remarkably stable over the time period of the study. It is difficult to generalize as to whether or not patients with more pharmaco-responsive pain would respond to tDCS in the same manner.

The identification of the appropriate motor cortex stimulation site was based on prior combined TMS pulse and EMG activity to map the cortex. We believe that this method of localization of motor cortex stimulation target is as accurate as possibly available and superior to that achieved by the alternative of the EEG 10/20 location system. The method described is similar to that reported in the recent study by Ihle et al,^35^ which showed no impact of tDCS of the motor cortex on pain processing in 16 healthy subjects.

### Interpretation

#### Recommendations for study design

Apart from recommending that a baseline pain score be included at the start of each treatment session, instead of at the start of the study alone, the study design could be implemented as per the study protocol.

#### Sample size calculation and recommendation for definitive study sample size

For calculation of a definitive sample size, we took the highest observed SD in our study for differences in pain scores between treatments, which was 0.67 (for sham vs cathode) at 14 days. The upper 95% CI limit for this SD was 0.99; therefore, we can use this as a conservative estimate of the SD. We set the minimum clinically important difference as a reduction of 15% of our observed mean pain score of 6.4 after 14 days of sham treatment). A sample size of 13 patients would give 90% power to detect a difference of 1 point between two treatments with α=0.05.

The overall sample size of this study was 24 subjects recruited and 21 followed through to the end of the study. This would suggest that this study is already large enough to detect a 15% change in pain scores in the overall trial but that sample size for the proportional analysis based on responder vs nonresponder subgroups has not been reached.

## Conclusion

As our pilot study was already larger than the calculated sample size for a definitive study, and our CI estimates indicate that all differences were significantly lower than the minimum clinically important difference of 1 point, the results of this pilot study do not support the need for a larger definitive study using the same stimulation parameters and the same electrode configuration.

Efforts have been directed toward better defining the precision of current application in tDCS, and advances in high-definition tDCS montage configurations may provide a more focal stimulation to the M1 cortex. Likewise, utilization of alternative parameters, such as transcranial alternating current stimulation or online remote confirmation of electrode placement, may enhance the design of future experiments. It does show that patients can quickly be acquainted with using these devices and home-based trials are both feasible and realistically achievable.

## Figures and Tables

**Table 1:** Individual patient characteristics

Subject ID	Sex	Diagnosis	Central or peripheral pain	Pain duration (months)	Response to rTMS	Pain medication

A	F	Spinal cord injury	Central	8	R	Gabapentin
B	M	Phantom limb pain	Peripheral	20	R	Gabapentin
C	M	Central post stroke pain	Central	9	R	None
D	M	Spinal cord injury	Central	10	R	None
E	M	CRPS type II	Peripheral	12	R	None
F	F	Phantom limb pain	Peripheral	3	NR	Gabapentin
G	M	Central post stroke pain	Central	11	R	None
H	M	Brachial plexus avulsion	Peripheral	13	NR	None
I	M	Central post stroke pain	Central	6	NR	Gabapentin
J	M	Central post stroke pain	Central	7	R	Pregabalin
K	M	Neuropathic pain right leg	Peripheral	13	R	Pregabalin
L	M	Central post stroke pain	Central	2	NR	None
M	F	Brachial plexus avulsion	Peripheral	4	NR	Pregabalin
N	F	Radiculopathy left hand	Peripheral	3	R	Pregabalin
O	M	Central post stroke pain	Central	11	NR	None
P	M	Trigeminal neuropathy	Peripheral	2	NR	Pregabalin
Q	F	Trigeminal neuropathy	Peripheral	20	NR	Pregabalin
R	F	Trigeminal neuropathy	Peripheral	7	NR	None
S	M	Central post stroke pain	Central	6	NR	None
T	F	Central post stroke pain	Central	4	R	Lamotrigene
U	F	Trigeminal neuropathy	Peripheral	5	R	Gabapentin
V	F	Central post stroke pain	Central	4	NR	Pregabalin
W	M	Neuropathic pain left arm	Peripheral	7	R	Pregabalin
X	M	Trigeminal neuropathy	Peripheral	6	R	Lamotrigene
Mean ±SD				(8.04±4.98)		

**Note:** Patients were classified as having neuropathic pain of either central or peripheral nerve origin based on diagnosis. Responders to rTMS had a reduction in pain intensity during the previous rTMS study.

**Abbreviations:** R, responder; NR, Non-responder; rTMS, repetitive transcranial magnetic stimulation.

**Table 2:** Number of stimulations delivered per trial completed for each subject

Subject ID	Anodal	Cathodal	Sham

A	0	0	5
B	5	5	5
C	5	5	5
D	0	0	5
E	5	5	4
F	5	5	5
G	4	5	5
H	5	5	7
I	5	5	5
J	5	5	6
K	5	5	5
L	5	5	5
M	5	5	5
N	0	0	5
O	5	5	5
P	3	4	5
Q	5	5	5
R	5	5	5
S	5	5	4
T	5	6	5
U	4	5	5
V	5	5	5
W	5	4	5
X	5	5	5

**Notes:** Target number of five stimulations per trial.

**Table 3:** Analysis of effect of tDCS on overall pain

Overall pain score										
	Anodal			Cathodal				Sham		
Day	N	Mean	SD	N	Mean	SD	N		Mean	SD
**Baseline**	20	6.43	1.71	21	6.19	1.68	23		6.48	1.83
**Day 1**	19	6.45	1.76	20	6.40	1.78	23		6.43	1.67
**Day 5**	18	6.53	1.70	21	6.43	1.73	23		6.70	1.53
**Day 14**	20	6.10	1.72	19	6.42	1.62	22		6.45	1.90

**Note:** No significant difference between baseline values of each treatment *P*=0.99 (one-way ANOVA) or in numerical rating scale mean values between Sham vs Anodal (–0.16, 95% CI: –0.43 to 0.11) *P*=0.43, Sham vs Cathodal (0.11, 95% CI: –0.16 to 0.37) *P*=0.94, or Cathodal vs Anodal (–0.27, 95% CI: –0.54 to 0.00) *P*=0.053.

**Table 4:** Adverse events for each type of stimulation for each individual subject.

Subject ID	Anodal	Cathodal	Sham	At Follow-up

A	None	None	None	None
B	Tiredness	Tiredness	Tiredness	None
C	None	None	None	Exacerbation of paresthesia in the affected area
D	None	None	Increase in pain	None
E	Tingling/redness	Tingling/redness	Tingling/redness	None
F	Tingling/redness	Tingling/redness	Tingling/redness	None
G	Headache	Headache	Headache	None
H	Tingling/redness	Tingling/redness	Tingling/redness	None
I	Tingling/redness	Tingling/redness	Tingling/redness	None
J	Tingling/redness/headache	Tingling/redness/headache	Tingling/redness/headache	None
K	None	None	None	None
L	Tingling/redness/headache	Tingling/redness/headache	Tingling/redness/headache	Tingling/redness/headache
M	Headache	Headache	Headache	None
N	None	None	Tingling/redness	None
O	Tingling	Tingling	Tingling	None
P	Tingling	Tingling	Tingling	None
Q	Tingling/redness	Tingling/redness	Tingling/redness	None
R	Tingling/tiredness	Tingling/tiredness	Tingling/tiredness	None
S	Tingling/redness	Tingling/redness	Tingling/redness	None
T	Tingling/redness	Tingling/redness	Tingling/redness	None
U	Tingling/redness	Tingling/redness	Tingling/redness	Exacerbation of paresthesia in the affected area
V	Tingling/redness	Tingling/redness	Tingling/redness	None
W	Tingling	Tingling	Tingling	None
X	Headache	Headache	Headache	None

**Notes:** Each of the three patients who reported symptoms at follow-up subsequently reported resolution within a further 3 months.

**Table 5:** Mean values and SDs of baseline and overall treatment on anxiety and depression scores

	Anodal			Cathodal			Sham		
Anxiety	N	Mean	SD	N	Mean	SD	N	Mean	SD
Baseline	21	7.14	2.61	21	7.38	3.34	23	7.48	2.97
Treatment	21	7.33	3.38	21	7.19	2.60	23	7.39	2.81
**Depression**	**N**	**Mean**	**SD**	**N**	**Mean**	**SD**	**N**	**Mean**	**SD**
Baseline	21	8.86	4.33	21	9.05	5.22	23	8.65	4.51
Treatment	21	8.90	5.05	21	9.14	4.50	23	8.70	4.32

**Notes:** No significant differences in anxiety or depression scores were demonstrated between baseline and at the end of each treatment period.
